# Prognostic and clinicopathological importance of microRNA-140 expression in cancer patients: a meta-analysis

**DOI:** 10.1186/s12957-021-02380-6

**Published:** 2021-09-03

**Authors:** Mengxia Zheng, Jingting Liu, Chunyan Meng, Kaifeng Tang, Jianhua Liao

**Affiliations:** 1 Department of General Surgery, Affiliated Zhejiang Hospital, Zhejiang University School of Medicine, 12 Lingyin Road, Zhejiang, 310013 Hangzhou China; 2 Department of Health Management, Sir Run Run Shaw International Medical Centre, 9 Jingtan Road, Zhejiang, 310000 Hangzhou China

**Keywords:** Cancer, MicroRNA-140, Meta-analysis, Prognosis, Biomarker

## Abstract

**Background:**

MicroRNA-140 (miR-140) is one of the most widely investigated miRNAs in cell carcinogenesis and cancer development. Despite present proposals of employing miR-140 as a candidate biomarker for cancer prognosis, its effectiveness in predicting patient survival and clinicopathological outcome is still under debate.

**Methods:**

A systematic search for English literature using online databases was performed with pre-established criteria. Odds ratios (ORs) or hazard ratios (HRs) with 95% confidence intervals (CIs) were collected to delineate the correlation between miR-140 levels and cancer patient prognosis.

**Results:**

For this meta-analysis, we selected 12 papers for analysis, involving 1386 participants. Based on our analysis, high levels of miR-140 were strongly correlated with enhanced patient overall survival (OS) (HR = 0.728, 95% CI = 0.601-0.882, *P* = 0.001). In addition, we also observed that elevated miR-140 levels significantly led to better OS in patients with cancers in different parts of the body like digestive system (HR = 0.675, 95% CI = 0.538-0.848, *P* = 0.001), digestive tract (HR = 0.709, 95% CI = 0.565-0.889, *P* = 0.003), and head and neck (HR = 0.603, 95% CI = 0.456-0.797, *P* < 0.001). Additionally, we verified that the low miR-140 levels was related to advanced TNM stage (OR = 0.420, 95% CI = 0.299-0.590, *P* < 0.001), worse histologic grade (OR = 0.410, 95% CI = 0.261-0.643, *P* < 0.001), and positive lymph node metastasis status (OR = 0.341, 95% CI = 0.144-0.807, *P* = 0.014).

**Conclusions:**

Taken together, our results suggest that elevated miR-140 levels can be employed as a favorable biomarker for cancer patient prognosis. This information can greatly benefit in the formation of an individualized therapeutic plan for the treatment of cancer patients.

## Background

MicroRNA-140 (miR-140) is one of the most widely investigated miRNAs in cell carcinogenesis and cancer development. Despite present proposals of employing miR-140 as a candidate biomarker for cancer prognosis, its effectiveness in predicting patient survival and clinicopathological outcome is still under debate. Here, we explored the association between miR-140 levels and clinicopathological features and patient prognosis in cancer by conducting an exhaustive review of relevant literature, with a goal to clarify the role of miR-140 in cancer.

## Introduction

Cancer is the most common noncommunicable disease and is expected to continue to rank as the leading cause of death worldwide [1]. Despite the advances in cancer treatment worldwide that have occurred in recent years and the improvements made in cancer prevention, diagnosis, surgery, and adjuvant therapy, cancer incidence and mortality are inexorably increasing worldwide. In addition to taking effective measures to improve the effects of aging and growth of the population and unequal socioeconomic development, researchers have carried out a large number of small and more in-depth studies to explore and mitigate the cancer burden [2]. To date, cancer-related lncRNAs, miRNAs, RNAs, and proteins have been continuously discovered to be related to prognosis after surgery [3–5]. However, paradoxical results have always appeared in previous studies; therefore, we conducted this analysis to find a stable prognostic marker that could potentially reduce the future cancer burden.

MicroRNAs (miRNAs), a class of conserved noncoding RNA 18-25 nucleotides in length, have attracted extensive attention in recent years and function in cancer pathogenesis through the cleavage or translational repression of targeted mRNAs [6]. As a small part of the entire human genome, miRNAs modulate levels of a considerable number of human genes, especially cancer-associated genes [7]. Meanwhile, a large quantity of miRNAs originates from genomic regions involved in cancer regulation. Moreover, miRNAs exert their biological role via regulation of cell proliferation, apoptosis, differentiation, and migration [8]. Indeed, emerging evidences suggest a strong relationship between aberrant miRNAs levels and various cancers. Among these miRNAs, miR-140 is one of the most widely investigated miRNAs in cell carcinogenesis and cancer development. It is well known that miR-140 is encoded within intron 16 of Wwp2 on the human chromosome 16 region and has a dual role in cancer progression [9]. On the one hand, miR-140 plays a tumor suppressive role in cancer. As revealed in published studies, suppression of cancer stem cell survival and invasive potential, cell cycle arrest, regulation of DNA synthesis and caspase 3/7 activity, control of NF-κB activity, and regulation of oncogenic protein expression correlate with miR-140 expression and miR-140 suppresses tumorigenesis by targeting various genes [10–12]. Alternately, Güllü et al. reported remarkably high expression of miR-140 in cancer tissues, which accelerates tumor progression [13]. Moreover, Meng et al. proposed that miR-140 modulates osteosarcomic chemoresistance via HMGN5 and autophagy regulation [14]. In addition, miR-140 is related to decreased levels of IGFBP-5 and involved in tamoxifen resistance in breast cancer [15]. Likewise, the clinical results of miR-140-related studies revealed similar findings as those of mechanistic studies. Therefore, given the importance of miR-140 in cancer, the exact function of miR-140 in different cancers should be further verified. However, thus far, there is no comprehensive analysis of miR-140 expression and its function in cancer patients.

Here, we explored the association between miR-140 levels and clinicopathological features (CPF) and patient prognosis in cancer by conducting an exhaustive review of relevant literature, with a goal to clarify the role of miR-140 in cancer.

## Materials and methods

### Search strategy and study selection

We carefully performed an exhaustive search for English publications using online databases, like PubMed, Embase, and the Cochrane Library from the inception of the databases to May 10, 2021. We employed keywords like “microRNA-140 OR miR-140” AND “cancer or tumor or malignancy or neoplasm or carcinoma” AND “prognosis or prognostic or survival or outcome.” Additionally, to conduct a thorough search of all relevant papers, we also scanned the references of all eligible papers to find publications that were missed in the previous search. Two authors separately reviewed all articles. A third author was available for discussion and resolution of any conflicts in data and conclusion. Details of the protocol for this systematic review were registered on INPLASY (INPLASY202180037) and are available in full on the inplasy.com (10.37766/inplasy2021.8.0037). This meta-analysis followed the strict guidelines of the reporting checklist that was included in the preferred reporting items for systematic reviews and meta-analyses statement [16].

### Study eligibility and ineligibility criteria

The following articles were included in our analysis: (1) all adult participants received a pathological diagnosis of cancer and received reasonable and effective therapeutic measures; (2) examination of miR-140 levels in cancerous tissues or blood; (3) all participants were separated into cohorts, based on their miR-140 levels, and survival analysis was completed on both cohorts; (4) sufficient data were provided to measure the hazard ratio (HR) and 95% confidence intervals (CI); and (5) studies involved more than 50 enrolled patients. Any articles that failed to comply with the above criteria were eliminated from our analysis. In addition, case reports, reviews, conference abstracts, letters, and animal trials were also excluded.

### Data accumulation and quality assessment

All data was separately compiled by two scientists. Relevant data included authors, publication year, research location, recruitment duration, population size, cancer type, detection procedure, detected sample, threshold, analysis type, HR prediction, and CPF. Study quality was assessed according to the Newcastle-Ottawa Quality Assessment Scale (NOS) [17].

### Statistical analysis

STATA 14.0 software (STATA Corporation, College Station, Texas, USA) was employed for all analyses. HRs and their subsequent 95% CIs were pooled to examine the association between miR-140 levels and the overall survival (OS) of cancer patients, and HRs from multivariate analyses in each study were preferentially included in the analysis. The relationship between miR-140 levels and CPF was examined by pooling the odds ratios (ORs) and their subsequent 95% CIs. The chi-square test and *I*^2^ statistic were employed to test heterogeneity. A fixed-effects model was used if the *P* value exceeded 0.05 and/or the *I*^2^ was less than 50%. Otherwise, the random-effects model was employed. Subgroup analyses were used to establish the relationship between miR-140 levels and cancer patient prognosis and to explore possible factors contributing to heterogeneity. Meanwhile, meta-regression was employed to further explore the heterogeneity among studies. Sensitivity analysis was carried out to confirm the stability of the accumulated consequences of miR-140 levels, in terms of the overall OS rate HR prediction. Begg’s and Egger’s tests analyzed publication bias. Two-sided *P* < 0.05 was significance threshold.

## Results

### Literature search and research characteristics

**Fig. 1:**
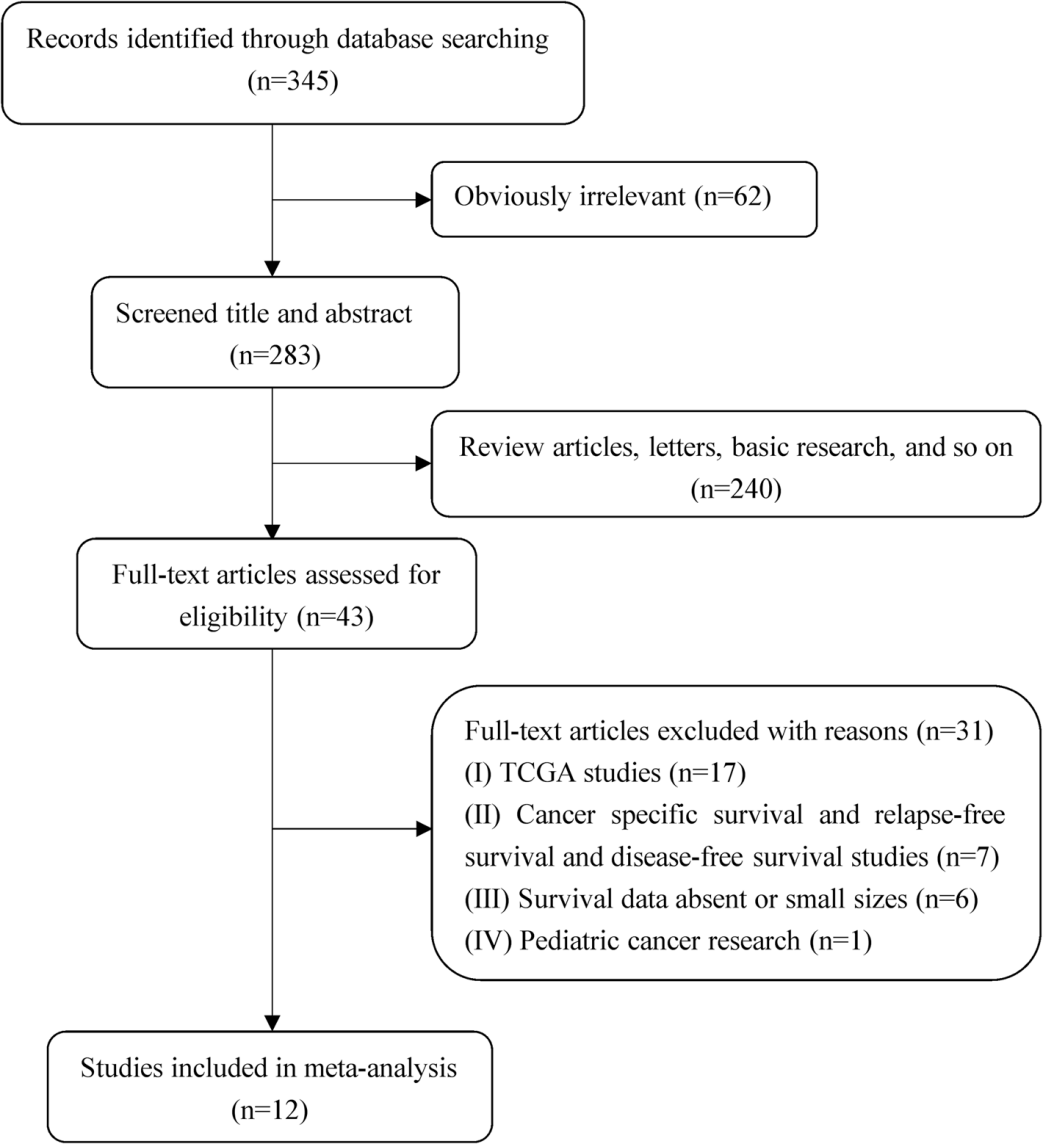
A summary of study eligibility and ineligibility process

The article eligibility process is summarized in Fig. 1. According to the prespecified keywords, we found 345 relevant articles in the initial search. After carefully reviewing the titles and abstracts, 302 of them were deemed ineligible since they were review articles, letters, basic research, etc. Then, we screened the remaining 43 studies for eligibility. Thirty-one of them were further excluded (TCGA studies [*n* = 17], cancer-specific survival and relapse-free survival and disease-free survival articles [*n* = 7], absence of survival data or small sample population [*n* = 6], pediatric cancer research [*n* = 1]). Finally, 12 articles covering 1386 patients from 2013 to 2020 were eligible for the meta-analysis. All patients were located in China and were diagnosed with different forms of cancer, including hepatocellular [18], gastric [19, 20], colorectal [21], nasopharyngeal [22–24], breast [25], cutaneous [26], esophageal squamous cell [27], thyroid [28], and renal cell [29]. MiR-140 levels were examined via quantitative real-time polymerase chain reaction (qRT-PCR) in 11 studies. Only one study used in situ hybridization. In most of the studies, miR-140 expression was measured in cancer tissues, whereas in three articles, it was measured in the serum. Six studies provided the median expression level of miR-140 as the cutoff value, and 6 studies used other optimal cutoff values. HRs were extracted from all of the included studies, of which 9 were postoperative and 3 were post-combined treatment. As listed in Table 1, the articles were of high quality, based on NOS assessment (quality score ≥ 6). More detailed information is presented in Table 1.

**Table 1:** Main characteristics of studies included in meta-analysis

Author	Year	Study region	Recruitment time	Sample size	Cancer type	Detection method	Detected sample	Cutoff scores (high/low)	Analysis method	OS, HR estimation	Quality score
Hao Y	2013	China	2004-2007	120	HCC	qRT-PCR	Tissues	Median	Univariate/multivariate analysis	0.47 (0.25-0.9)	8
Fang Z	2017	China	NS	144	GC	ISH	Tissue	Optimal cutoff	Univariate/multivariate analysis	0.505 (0.319-0.801)	7
Li J	2017	China	NS	63	CRC	qRT-PCR	Blood	Median	Univariate analysis	0.77 (0.65-0.92)	6
Cha Y	2018	China	2011-2015	60	GC	qRT-PCR	Tissue	Median	Univariate analysis	0.39 (0.17-0.93)	7
Zhou Y	2019	China	2010-2012	73	BC	qRT-PCR	Tissue	Median	Univariate analysis	0.79 (0.65-0.96)	7
Zhang H	2020	China	2014-2016	200	NPC	qRT-PCR	Blood	Median	Univariate analysis	1.04 (0.41-2.62)	6
Zou X	2020	China	2014-2016	208	NPC	qRT-PCR	Blood	Median	Univariate analysis	0.85 (0.37-1.98)	6
He Y	2020	China	NS	104	CM	qRT-PCR	Tissue	Optimal cutoff	Univariate/multivariate analysis	0.8 (0.39-0.92)	7
Yang H	2020	China	1998-2003	113	ESCC	qRT-PCR	Tissues	Optimal cutoff	Multivariate analysis	0.84 (0.67-1.05)	8
Yu Q	2020	China	2009-2013	122	TC	qRT-PCR	Tissues	Optimal cutoff	Multivariate analysis	0.369 (0.152-0.898)	8
Wu Q	2020	China	2013-2018	105	NPC	qRT-PCR	Tissues	Optimal cutoff	Univariate analysis	0.57 (0.41-0.8)	7
Huang C	2020	China	NS	74	RCC	qRT-PCR	Tissues	Optimal cutoff	Multivariate analysis	1.16 (1.01-1.33)	7

*HCC* hepatocellular carcinoma, *GC* gastric cancer, *CRC* colorectal cancer, *BC* breast cancer, *NPC* nasopharyngeal carcinoma, *CM* cutaneous melanoma, *ESCC* esophageal squamous cell carcinoma, *TC* thyroid cancer, *RCC* renal cell carcinoma, *qRT-PCR* quantitative real-time PCR, *ISH* in situ hybridization, *NS* data were not shown, *OS* overall survival, *HR* hazard ratio

### Significance of miR-140 expression in the prognosis of cancer patients

**Fig. 2:**
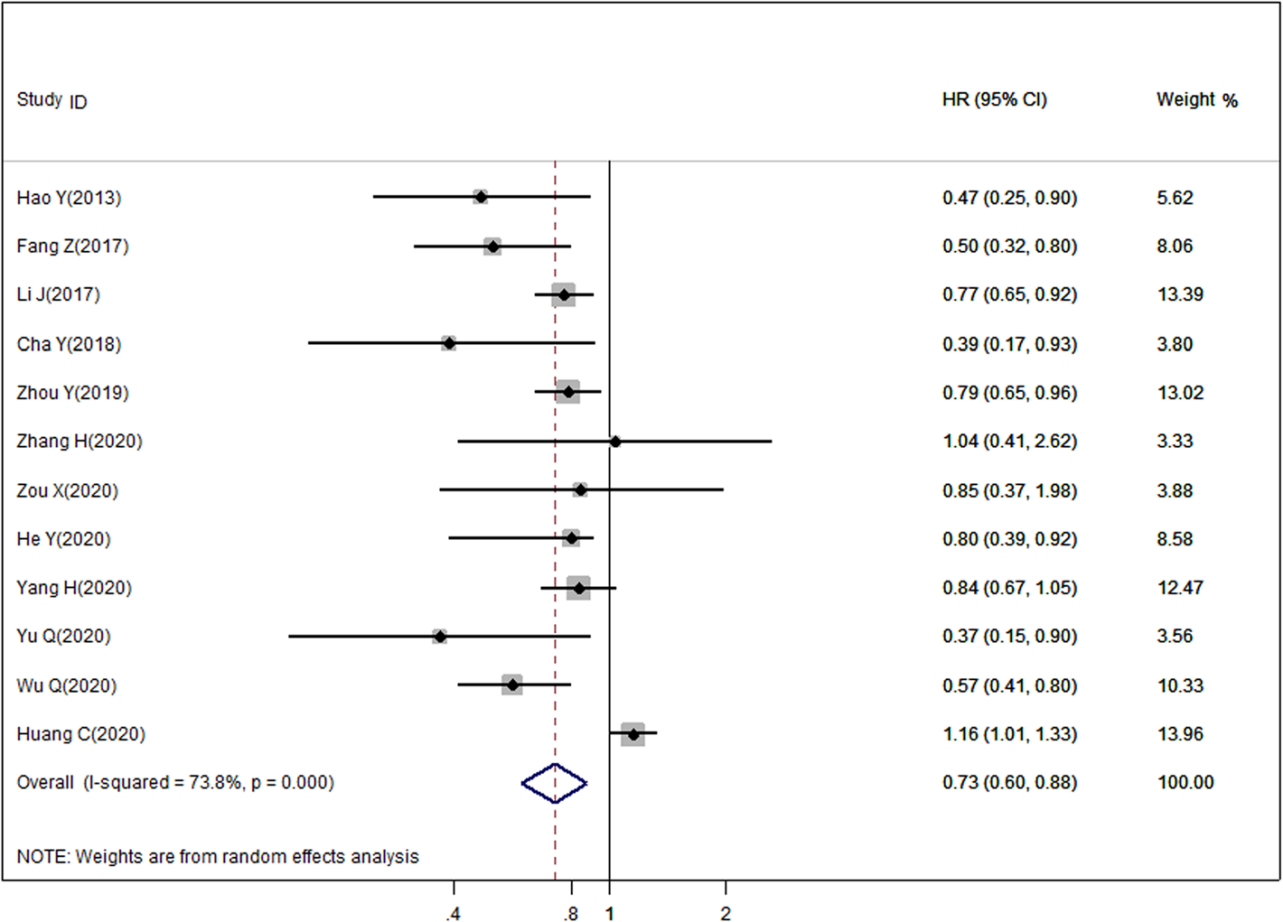
Forest plot illustrating relationship between miR-140 levels and overall survival (OS) rate of cancer patients

As indicated in Table 2, we demonstrated that elevated miR-140 levels in cancer was significantly correlated with enhanced patient OS than low miR-140 expression (HR = 0.728, 95% CI = 0.601-0.882, *P* = 0.001, Fig. 2). Combined analysis also revealed that upregulated miR-140 levels were correlated with increased OS in both the postoperative (HR = 0.734, 95% CI = 0.594-0.907, *P* = 0.004) and nonpostoperative groups (HR = 0.636, 95% CI = 0.474-0.854, *P* = 0.003). Specifically, we revealed that high miR-140 levels markedly enhanced OS in patients with digestive system cancer (HR = 0.675, 95% CI = 0.538-0.848, *P* = 0.001), digestive tract cancer (HR = 0.709, 95% CI = 0.565-0.889, *P* = 0.003), and head and neck cancer (HR = 0.603, 95% CI = 0.456-0.797, *P* < 0.001). In addition, the pooled HR suggested a strong association between miR-140 levels and OS in the univariate (HR = 0.727, 95% CI = 0.652-0.809, *P* < 0.001) and multivariate (HR = 0.716, 95% CI = 0.518-0.990, *P* = 0.043) subgroup, as well as in the tissue miR-140 (HR = 0.688, 95% CI = 0.539-0.880, *P* = 0.003) and blood miR-140 (HR = 0.781, 95% CI = 0.660-0.923, *P* = 0.004) subgroups. In the analysis stratified by cutoff value, we found that high miR-140 expression was still a predictor of OS in the median level (HR = 0.759, 95% CI = 0.671-0.858, *P* < 0.001) and optimal cutoff value (HR = 0.721, 95% CI = 0.525-0.989, *P* = 0.043) subgroups. Furthermore, this correlation was confirmed in the subgroup analysis based on publication date (< 5 years, HR = 0.773, 95% CI = 0.615-0.973, *P* = 0.028; ≥ 5 years, HR = 0.618, 95% CI = 0.427-0.876, *P* = 0.007, respectively). Moreover, a marked difference was also observed involving > 100 patients (HR = 0.699, 95% CI = 0.602-0.811, *P* < 0.001) but not in studies with smaller sizes (HR = 0.832, 95% CI = 0.623-1.112, *P* = 0.215).

**Table 2:** Meta-analysis of miR-140 expression and prognosis in cancers

Categories	Studies (patients)	HR (95% CI)	*I*^**2**^ (%)	*P*_h_	*Z*	*P*
OS	12 (1386)	0.728 (0.601-0.882)	73.8	< 0.001	3.25	0.001
Postoperative OS	9 (873)	0.734 (0.594-0.907)	77.6	< 0.001	2.86	0.004
Non-postoperative OS	3 (513)	0.636 (0.474-0.854)	0.0	0.377	3.01	0.003
Cancer type						
Digestive system cancer	5 (500)	0.675 (0.538-0.848)	51.1	0.085	3.37	0.001
Digestive tract cancer	4 (380)	0.709 (0.565-0.889)	51.4	0.104	2.97	0.003
Head and neck cancer	4 (635)	0.603 (0.456-0.797)	7.8	0.354	3.55	< 0.001
Analysis method						
Multivariate analysis	6 (677)	0.716 (0.518-0.990)	80.8	< 0.001	2.02	0.043
Univariate analysis	9 (1077)	0.727 (0.652-0.809)	16.9	0.292	5.81	< 0.001
Publication date						
< 5 years	9 (1059)	0.773 (0.615-0.973)	73.5	< 0.001	2.19	0.028
≥ 5 years	3 (327)	0.618 (0.427-0.876)	55.9	0.104	2.71	0.007
Size						
< 100	4 (270)	0.832 (0.623-1.112)	85.8	< 0.001	1.24	0.215
> 100	8 (1116)	0.699 (0.602-0.811)	34.4	0.153	4.73	< 0.001
Cutoff value						
Median	6 (724)	0.759 (0.671-0.858)	4.1	0.391	4.39	< 0.001
Optimal cutoff	6 (662)	0.721 (0.525-0.989)	83.5	< 0.001	2.03	0.043
Detected sample						
Tissue	9 (915)	0.688 (0.539-0.880)	80.0	< 0.001	2.98	0.003
Blood	3 (471)	0.781 (0.660-0.923)	0.0	0.806	2.90	0.004

*OS* overall survival, *HR* hazard ratio, *CI* confidence interval, *P*_h_ *P* value for heterogeneity based on *Q* test, *P P* value for statistical significance based on *Z* test

The heterogeneity of the OS rate in the included datasets was remarkable as determined by the chi-square test and *I*^2^ statistic (*I*^2^ = 73.8%, *P*_h_ < 0.001); thus, we employed a random-effects model to measure the HR and 95% CI. Moreover, subgroup analysis and meta-regression examined the possible origin of heterogeneity in our analysis, however, it showed that the heterogeneity was obvious in most stratified analyses, and the heterogeneity was not a result of therapy method (*P* = 0.805), cancer type (*P* = 0.927), analysis method (*P* = 0.330), publication date (*P* = 0.233), sample size (P = 0.247), cutoff value (*P* = 0.897) or detected sample (*P* = 0.176).

### Correlation between miR-140 levels and CPF of cancer

In 7 articles, data regarding the relationship between miR-140 levels and the CPF of cancer were reported. A summary of these articles is provided in Table 3. The pooled OR indicated a marked correlation between miR-140 expression and TNM stage (OR = 0.420, 95% CI = 0.299-0.590, *P* < 0.001), histologic grade (OR = 0.410, 95% CI = 0.261-0.643, *P* < 0.001), and lymph node (LN) metastasis (OR = 0.341, 95% CI = 0.144-0.807, *P* = 0.014), suggesting that low levels of miR-140 was proportional to advanced TNM stage, worse histologic grade, and positive LN metastasis status. However, miR-140 expression was not correlated with age (OR = 1.068, 95% CI = 0.761-1.499, *P* = 0.703), sex (OR = 0.859, 95% CI = 0.500-1.475, *P* = 0.582), or tumor size (OR = 1.152, 95% CI = 0.352-3.773, *P* = 0.815).

**Table 3:** Meta-analyses of miR-140 expression classified by clinicopathological parameters

Study covariates	Studies (patients)	OR (95% CI)	*I*^**2**^ (%)	*P*_h_	Z	*P*	Model
Gender (male/female)	7 (770)	1.068 (0.761-1.499)	30.0	0.199	0.38	0.703	Fixed
Age (< 60/≥ 60)	3 (257)	0.859 (0.500-1.475)	0.0	0.519	0.55	0.582	Fixed
Tumor size (≤ 5/> 5 cm)	3 (257)	1.152 (0.352-3.773)	79.0	0.008	0.23	0.815	Random
TNM stage (1-2/3-4)	6 (614)	0.420 (0.299-0.590)	43.4	0.113	5.00	< 0.001	Fixed
LN metastasis (absence/presence)	6 (568)	0.341 (0.144-0.807)	78.9	< 0.001	2.45	0.014	Random
Histologic grade (well+ moderately differentiated/ poorly differentiated)	4 (389)	0.410 (0.261-0.643)	0.0	0.552	3.88	< 0.001	Fixed

*LN* lymph node, *OR* odds ratio, *CI* confidence intervals, *P*_h_ *P* value for heterogeneity based on *Q* test, *P P* value for statistical significance based on *Z* test

### Sensitivity analysis and publication bias

**Fig. 3:**
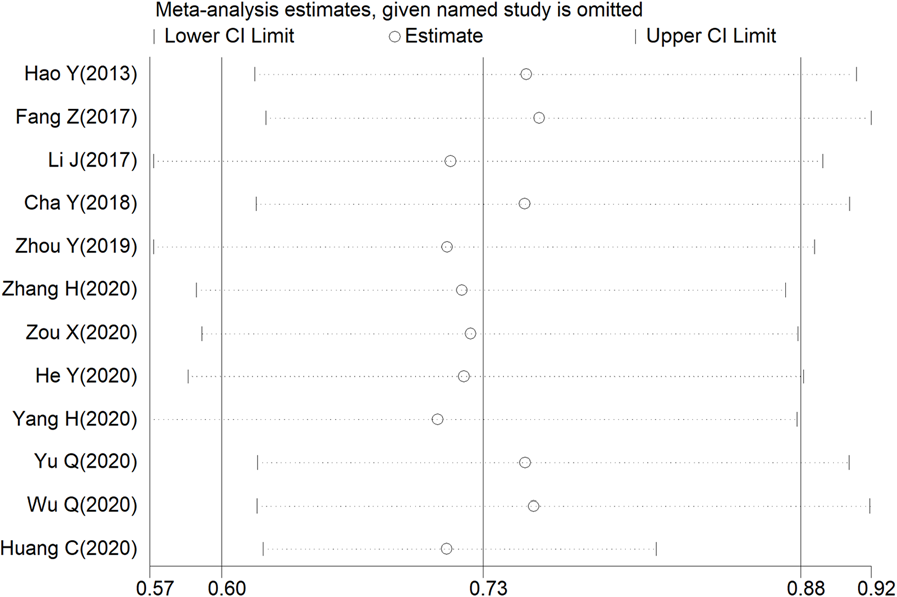
Sensitivity analysis of overall survival (OS) rates for cancer patients

**Fig. 4:**
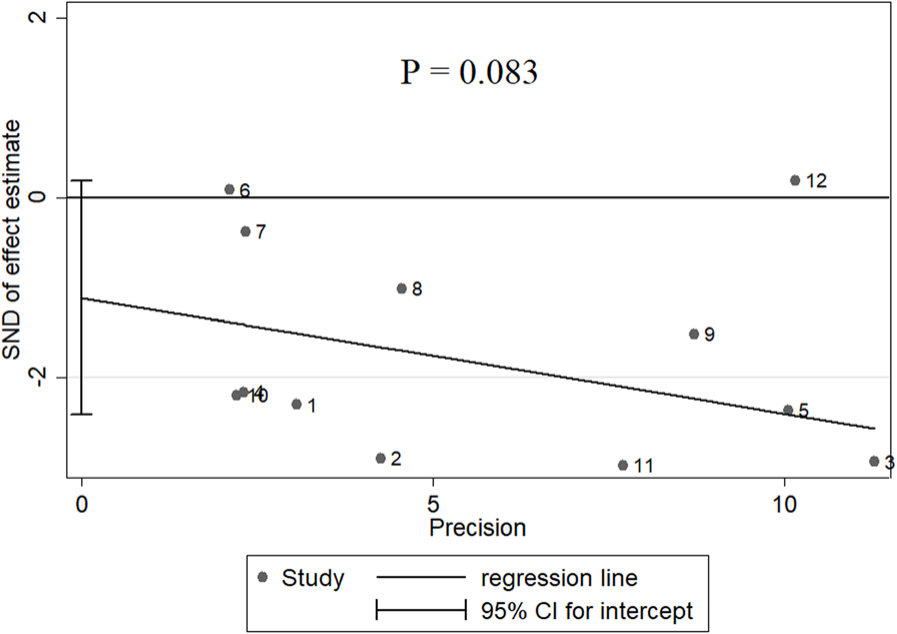
Effect estimate of publication bias of selected articles, using the Egger’s test

To gain insights into the stability of this meta-analysis, further sensitivity analysis was performed. As shown in Fig. 3, each point of the omitted single dataset is estimated to be within the 95% CI, indicating that the meta-analysis results were not dominated by one single study. Moreover, we used Egger’s (*P* = 0.083) and Begg’s tests (*P* = 0.371) to assess publication bias in the eligible articles. As shown in Fig. 4, no discernible publication bias was present.

## Discussion

Unhealthy living habits, the intake of toxic substances, and the aging of the population inevitably increases the cancer burden, and marked geographic and economic diversity still exist in cancer prevalence. The mitigation of these factors requires the broad community participation, which reminds us of the recent global assessment of cancer that indicated the need to tailor cancer control mechanisms to local conditions. In fact, the incidence and mortality of cancer have been increasing yearly. Given that the environment cannot be improved in a short time period, researchers have committed to discovering mechanisms underlying the occurrence and development of cancer and finding treatment breakthroughs. Fortunately, in recent years, new advances in the knowledge of genes and proteins and the development of surgical procedures and adjuvant therapies have, to a certain extent, reduced the impact of cancer [30–32]. Based on the current research focus on miRNAs, this we aimed to find a therapeutic direction to further improve the prognosis of tumor patients.

MiRNAs constitute a collection of noncoding RNAs with important impacts on gene expression patterns [6]. As miR-140 is one of the most widely investigated miRNAs, its role in the pathophysiology of neoplastic miRNAs has been comprehensively assessed [33]. However, the role of altered miR-140 levels and function in cancer is still controversial. Recently, diminished expression of miR-140 in cancer tissue has been revealed in numerous cancers. In a previous report, miR-140 was shown to be sponged by circ-ATAD1 during cancer progression. Additionally, its suppression of the epithelial-mesenchymal transition was impaired by the oncogenic long noncoding RNA, namely, TMPO-AS1 [34, 35]. In addition, overexpression of miR-140-5p could inhibit proliferation, migration, and invasion and promote apoptosis in cancer by downregulating MAPK1, TRIM28, and YES1, further regulating levels of cleaved caspase-3, Bcl-2, and Bax, and blocking nuclear transport and Wnt/β-catenin signaling [19, 20, 25, 36, 37]. Emerging studies also showed that low miR-140 levels is closely related to elevated MALAT1 and PAK1 levels [38]. Functionally, miR-140 not only targets oncogene loci but could also be involved in the TLR4/NF-κB axis and KCNQ1OT1/miR-140-5p/SOX4 axis [39, 40]. Although miR-140 is widely recognized as a tumor suppressor, the suggestion that miR-140 is an oncogenic agent continues to emerge. For instance, miR-140 is highly expressed in certain cancers and promotes cell proliferation, migration, and invasion via its regulation of autophagy and KLF9 levels [29]. Increased expression of miR-140 could modulate cancer cell chemoresistance by targeting IGFBP-5 and sensitizes osteosarcoma cells to chemotherapy by promoting HMGN5-mediated autophagy [14, 15]. In addition, the results of clinical utility studies on miR-140 were also varied [18, 22, 29]. Based on these findings, we specifically performed this meta-analysis to examine the prognostic ability of miR-140 levels in cancer.

Here, we selected 12 articles involving 1386 patients. Our data demonstrated that low miR-140 levels are strongly correlated with worse OS in cancer patients. Specifically, the prognostic capability of miR-140 levels was further verified in patients with digestive system cancer, digestive tract cancer, and head and neck cancer. Moreover, this finding was observed in patients with both surgically and nonsurgically treated cancer. Interestingly, it can be seen from the stratified analysis that a majority of the analyses confirmed the prognostic capability of miR-140. Hence, miR-140 expression may be a stand-alone biomarker for cancer patient prognosis. We found significant OS rate heterogeneity among the included studies; however, factors such as therapy method, cancer type, analytic design, publication date, sample population, threshold, and detected sample were not the source of this heterogeneity. Therefore, to eliminate the influence of heterogeneity to some extent, we used a random-effects model to compute the HR and 95% CI where necessary. On the other hand, the results of this meta-analysis indicated that low miR-140 levels were correlated with advanced TNM stage and worse histologic grade and positive LN metastasis in cancer patients. Compared with patients with augmented miR-140 levels, reduced miR-140 levels were strongly associated with advanced tumor grade. Herein, we hypothesize that a low level of miR-140 expression induces tumor progression via several pathways, thus contributing to poor cancer patient prognosis. This is a novel meta-analysis on the clinicopathology and prognosis of miR-140 expression, and our findings can be used to guide subsequent studies related to miR-140 expression.

Even though our analysis demonstrated valid evidence of the excellent prognostic capacity of miR-140 expression, the limitations associated with this study cannot be ignored. First, 9 different tumor types were analyzed, but only 12 studies were included. Given the low number of studies, the analysis may be unconvincing. Second, different cutoff values of miR-140 expression across studies may have influenced the results. Third, all of the articles were from China, which limits the applicability of the results to other populations. Fourth, some HRs and CIs were computed from survival curves, which inevitably introduced statistical errors. Finally, the level of heterogeneity among the studies was relatively significant and, unfortunately, we did not find the source. Thus, to draw a more convincing conclusion on the clinical utility of miR-140 in cancer patients, additional investigations, involving multiple cancer types, with numerous patients and appropriate and unified methods, are needed worldwide.

## Conclusion

In summary, our meta-analysis, which synthesized the results of all eligible studies, showed that low expression of miR-140 led to worse OS, advanced TNM stage, worse histologic grade, and positive LN metastasis status in cancer patients. Therefore, we have reason to believe that miR-140 is not only a stand-alone indicator of patient survival but may also become a new target for cancer therapy.

## Data Availability

All data are available from the corresponding author.

## Abbreviations

MiR-140: MicroRNA-140
ORs: Odds ratios
HRs: Hazard ratios
CIs: Confidence intervals
OS: Overall survival
CPF: Clinicopathological features
NOS: Newcastle-Ottawa Quality Assessment Scale
LN: Lymph node
